# A Novel High Content Imaging-Based Screen Identifies the Anti-Helminthic Niclosamide as an Inhibitor of Lysosome Anterograde Trafficking and Prostate Cancer Cell Invasion

**DOI:** 10.1371/journal.pone.0146931

**Published:** 2016-01-19

**Authors:** Magdalena L. Circu, Samantha S. Dykes, Jennifer Carroll, Kinsey Kelly, Floyd Galiano, Adam Greer, James Cardelli, Hazem El-Osta

**Affiliations:** 1 Feist-Weiller Cancer Center, Louisiana State University Health Sciences Center, Shreveport, Louisiana, United States of America; 2 Department of Microbiology and Immunology, Louisiana State University Health Sciences Center, Shreveport, Louisiana, United States of America; The Chinese University of Hong Kong, HONG KONG

## Abstract

Lysosome trafficking plays a significant role in tumor invasion, a key event for the development of metastasis. Previous studies from our laboratory have demonstrated that the anterograde (outward) movement of lysosomes to the cell surface in response to certain tumor microenvironment stimulus, such as hepatocyte growth factor (HGF) or acidic extracellular pH (pHe), increases cathepsin B secretion and tumor cell invasion. Anterograde lysosome trafficking depends on sodium-proton exchanger activity and can be reversed by blocking these ion pumps with Troglitazone or EIPA. Since these drugs cannot be advanced into the clinic due to toxicity, we have designed a high-content assay to discover drugs that block peripheral lysosome trafficking with the goal of identifying novel drugs that inhibit tumor cell invasion. An automated high-content imaging system (Cellomics) was used to measure the position of lysosomes relative to the nucleus. Among a total of 2210 repurposed and natural product drugs screened, 18 “hits” were identified. One of the compounds identified as an anterograde lysosome trafficking inhibitor was niclosamide, a marketed human anti-helminthic drug. Further studies revealed that niclosamide blocked acidic pHe, HGF, and epidermal growth factor (EGF)-induced anterograde lysosome redistribution, protease secretion, motility, and invasion of DU145 castrate resistant prostate cancer cells at clinically relevant concentrations. In an effort to identify the mechanism by which niclosamide prevented anterograde lysosome movement, we found that this drug exhibited no significant effect on the level of ATP, microtubules or actin filaments, and had minimal effect on the PI3K and MAPK pathways. Niclosamide collapsed intralysosomal pH without disruption of the lysosome membrane, while bafilomycin, an agent that impairs lysosome acidification, was also found to induce JLA in our model. Taken together, these data suggest that niclosamide promotes juxtanuclear lysosome aggregation (JLA) via modulation of pathways involved in lysosome acidification. In conclusion, we have designed a validated reproducible high-content assay to screen for drugs that inhibit lysosome trafficking and reduce tumor invasion and we summarize the action of one of these drugs.

## Introduction

Lysosomes are multifunctional intracellular organelles containing hydrolytic enzymes that degrade macromolecules and cellular components [1]. Lysosomes were classically thought to only function in cellular housekeeping, but recent evidence suggests that these organelles also contribute to the pathology of many clinically relevant diseases, including malignancies. Lysosomes are involved in tumorigenesis through many different mechanisms, including dysregulated autophagy, aberrant lysosomal trafficking and exocytosis, and increased lysosome membrane permeabilization (LMP) [2,3]. Due to the wide variety of lysosome-mediated functions that play a role in tumor survival, lysosomes have recently been gaining attention as an attractive target for cancer therapeutics.

Formation of metastatic colonies resulting from an invasive primary tumor is the leading cause of cancer-related deaths. Unfortunately there are no available drugs that inhibit this process. Therefore, increased understanding of invasion is urgently needed in order to develop effective therapies to prevent tumor progression. Our previous studies demonstrate that lysosome trafficking plays an important role in regulating cancer cell invasion, whereby tumor cells with lysosomes located close to the plasma membrane secrete more proteases and are more invasive than cells with lysosomes clustered in the perinuclear region [4–7]. Particularly, we have shown that several common features of the solid tumor microenvironment, hepatic growth factor (HGF), and acidic extracellular (pHe) trigger lysosome outward movement, accompanied by increased cathepsin B secretion and tumor cell invasion.

Indeed, cathepsin B is a lysosomal cysteine protease that plays a role in protein turnover within lysosomes [8]. In malignant cells, the expression of cathepsin B is highly up regulated compared to normal tissue, and the enzyme can be found within actin rich invasive protrusions termed invadopodia [9,10]. Evidence supports the notion that lysosomal proteases, including cathepsin B, are secreted into the extracellular environment, where these proteases participate in the degradation of the extracellular matrix (ECM), a necessary event in cancer cell invasion [9,10].

Lysosomes move along microtubules and actin filaments via association with molecular motor proteins, including dyneins, kinesin and myosin [11–13]. Moreover, several GTPases including RhoA, Rab7, and Rab27 recruit motor proteins to lysosomes, thus providing strict regulation of lysosome motility throughout the cell [14–16]. Notably, the minus-end-directed movement of lysosomes along the microtubules is dependent on Rab7 and Rab interacting lysosomal protein (RILP), which recruit dynein motors to lysosomes and promotes retrograde transport [12]. In this regard, inhibiting anterograde lysosome trafficking by overexpression of RILP results in reduced levels of secreted cathepsin B and tumor cell invasion [4]. Interestingly, Troglitazone (Tro) and 5-(N-ethyl-N-isopropyl)-amiloride (EIPA), sodium proton exchanger inhibitors, promote retrograde trafficking of peripheral lysosomes in prostate cancer cells, resulting in reduced protease secretion and tumor cell invasion [4]. EIPA and Tro-mediated juxtanuclear lysosome aggregation (JLA) is Rab7/RILP dependent.

Another factor that may contribute to lysosome trafficking is the Vacuolar type proton-ATPase pump (V-ATPase). This large enzymatic complex transforms the energy of ATP hydrolysis into the movement of protons across lysosomal membrane. In addition to generating lysosomal acidification, V-ATPase is also implicated in other cellular functions including vesicular trafficking. Indeed, the c-subunit has been shown to interact with Arf6, a small GTPase that directs membrane trafficking and cytoskeletal dynamics [17]. In osteoclasts, ATP6AP1 (a subunit of V-ATPase also known as Ac45) interacts with the small GTPase Rab7, which regulates vesicular trafficking [18]. The A-subunit isoform is also believed to be crucial in vesicular trafficking [19].

As previously characterized inhibitors of anterograde lysosome trafficking, such as Tro and EIPA cannot be advanced into the clinical arena [20], we sought to identify additional novel repurposed and natural product compounds many of which are already in clinical use for non-cancer indications. Our goal was to identify novel inhibitors of anterograde lysosome trafficking with a good safety profile. This represents a novel approach in translational cancer research that could potentially discover new effective anti-invasion and anti-metastatic drugs.

In this manuscript, we present the results of a screen of 4 libraries of compounds, some of which are already marketed for non-cancer indications, utilizing a novel high content approach combining fluorescence microscopy and multi-parameter image analysis. We identified several drugs that inhibit anterograde lysosome trafficking, including niclosamide. Niclosamide (C13H8Cl2N2O4, MW 327) is an FDA-approved oral anti-helminthic agent, widely available outside the US for the treatment of intestinal tapeworms. It is inexpensive, generally well tolerated and associated with few side effects [21]. Niclosamide exerts its toxic effect against helminthes by uncoupling oxidative phosphorylation [22,23] and has lately been shown to possess a promising effect against cancer. In this report, we investigated niclosamide’s mechanism of action and effect on lysosomes protease secretion, cancer cell motility, and invasion.

## Material and Methods

### Cell lines and culture

The human prostate cancer cell line DU145 and human glioma cell line A172 were obtained from American Type Culture Collection (Manassas, VA). DU145 cells were maintained in RPMI-1640 (Mediatech, Corning, NY) supplemented with 10% Fetal Bovine Serum (FBS) and 1% penicillin-streptomycin. A172 glioma cell lines were maintained in Dulbeco Modified Eagle’s Medium (DMEM) (Mediatech) supplemented with 10% FBS. Both cell lines were maintained in a 37°C incubator with 5% CO2 and were passaged upon attaining more than 75% confluence. Buffered RPMI-1640 was prepared from RPMI-1640 powder supplemented with 10mM NaHCO_3_ and 20mM NaCl and adjusted to pHe 6.4.

### High content screen for inhibitors of peripheral lysosome trafficking

**Fig 1:**
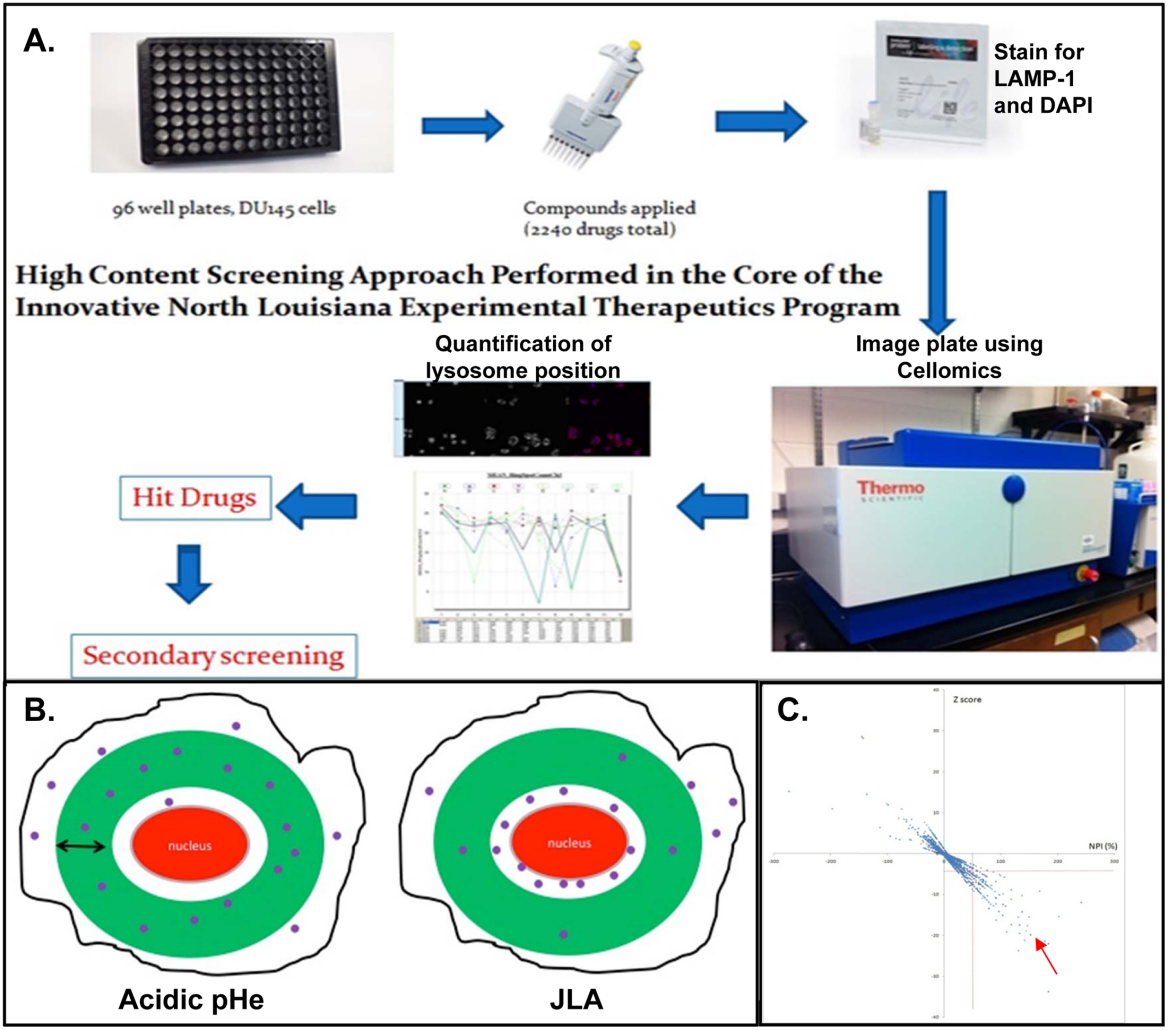
A high content imaging approach was used to identify compounds that cause JLA. **(A)** The methodology of the cellomics-based high content screening approach. **(B)** DAPI staining is used by the cellomics imaging platform to identify individual cells. A virtual mask is drawn by the machine (presented by a green ring) with a predetermined width and distance from the nucleus. The number of lysosomes inside the mask is calculated by the machine (represented by the purple spot inside the ring). By treating cells with acidic pHe, lysosomes move out and the number of spots inside the ring increases. By inhibiting lysosome movement, the number of spots around the nucleus increases whereas the number of spots inside the ring mask decreases. This property was used to calculate relative lysosome distribution. **(C)** The functional effect for each compound relative to control was calculated using “normalized percent of inhibition” (NPI). Statistical significance between compounds and the negative control was measured by Z score. A scatter plot representing the NPI (X-axis) and the Z score (Y-axis) of each compound is drawn. The drugs that yield an NPI score of more than 50% and a Z score of less than -4 (right lower quadrant as indicated by the red arrow) were designated as ‘hit’ compounds.

DU145 cells were seeded in 96 well plates at 4,500 cells per well. RPMI buffered media titrated at pH of 6.4–6.8 was added to column 12 after the media was removed and serves as negative control. Compounds to be screened were added to column 2–11 after dilution in low pH buffered media at a final concentration of 5uM. The 4 compound libraries included in the current screen include the NIH Clinical Collection (450 drugs), Prestwick (1200 drugs), phytochemical (320 drugs) and GreenPharma (240 drugs). 25 μM EIPA served as a positive control. After a 16 hour incubation, cells were fixed with 4% cold paraformaldehyde (Sigma-Aldrich, St. Louis, MO) for 20 minutes. Cells were washed with phosphate buffered saline (PBS) then lysosomes were stained by incubation with the H4A3 LAMP-1 antibody diluted at 1:200 in 0.25% BSA and 0.1% saponin in PBS (BSP) for 1 hour. Cells were then washed with PBS 3 times and incubated for 1 hour with Dylight Donkey anti-mouse diluted at 1:200 in BSP. Cells were then washed with PBS 3 times and incubated for 20 minutes with DAPI (Sigma-Aldrich) diluted at 1:1000 in PBS. DAPI was washed off with PBS and cells were maintained in PBS for the duration of the screening process. Plates were mounted and read in a Cellomics Arrayscan (Thermo Fisher Scientific, Inc, Waltham, MA) automated fluorescence imager (Fig 1A). Cells were photographed using 20X objective in 2 fluorescent channels. A total of 15 different fields in each well, and a maximum of 300 cells were imaged per well. The biocompartmental analysis algorithm was used to identify each cell by its nucleus and to apply a cytoplasmic mask (ring) and calculate the number of lysosomes inside the ring. The ring was of 12 pixels width, and its inner rim was 6 pixels away from the nucleus. The average number of lysosomes inside the designated ring region was acquired as “mean ring spot count channel 3”, which represents the distribution of lysosomes relative to the nucleus. When lysosomes move away from the nucleus and disperse throughout the cytoplasm, the number of spots representing lysosomes inside the cytoplasmic mask increases. By contrast, lysosomes cluster near the nucleus the numbers of spot inside the cytoplasmic ring decreases (Fig 1B). The Z’ factor of the assay was determined from the EIPA (positive control) and low pH (negative control) [24]. Experiments were performed in duplicate. Only plates with positive Z’ factor were used for analysis.

### Reagents and antibodies

LY294002, U0126, Bafilomycin A and nocodazole were purchased from Calbiochem (San Diego, CA) and were used at 10 μM, 10 μM, 100nM and 10 μM, respectively. Phalloidin 1:200 was purchased from Life Technologies (Grand Island, NY). The α-tubulin antibody, 1:1000, was purchased from Neomarkers (Fremont, CA). LAMP1 antibody (H4A3), 1:200, was purchased from the Developmental Studies Hybridoma bank at the University of Iowa. DyLight 594 or FITC, donkey anti-mouse or anti-rabbit were purchased from Jackson Immunoresearch (West Grove, PA). pAKT and pMet were used at 1/1000 and purchased from Cell Signaling (Beverly, MA). EIPA (25 μM), cytochalasin D (1 μM), Rab7 antibody (1:1000), and chloroquine (50 μM) were purchased from Sigma-Aldrich (St. Louis, MO). Secondary HRP-conjugated anti-rabbit and anti-mouse were purchased from GE Healthcare (Pittsburgh, PA).

### Immunofluorescence

Cells were seeded at 50–70% confluence on glass coverslips. Cells were fixed with 4% paraformaldehyde (PFA) for 20 minutes. Cells were then washed with PBS and incubated with primary antibody (1:200 diluted in BSP) for one hour. Cells were then washed again with PBS prior to incubation with fluorescently conjugated secondary antibody (1:200 diluted in BSP) for one hour. For actin staining, cells were incubated with phalloidin diluted at 1:200 in BSP for 20 minutes. Slides were mounted using Slowfade Anti-Fade Gold reagent with DAPI (Life Technologies, Grand Island, NY). Images were acquired using an Olympus UPlanFL 40X/0.75 objective and an Olympus BX50 microscope with MetaMorph software. Images were merged using imageJ software. For confocal images, a HCX Plan Apo 63X/1.4–0.6 oil objective on the Leica TCS SP5 microscope and Leica LAS AF software were used.

### Acridine orange staining

Cells grown on glass cover slips were loaded with 10 μg/mL acridine orange (Sigma Aldrich) for 20 minutes at 37°C then washed with PBS. Following an overnight incubation with different compounds, cells were fixed with cold 4% PFA for 20 minutes and then slides were mounted with Slowfade Anti-Fade Gold Reagent with DAPI, prior to visualization by immunofluorescence microscopy.

### Proliferation assay

Cells were seeded in 96-well plate at 4,500 cells per well and grown for 16 hours. Numbers of viable cells in triplicate wells were determined after CellTiter-Blue^®^ (Promega, Madison, WI) reagent was added to each well at 1/5 volume ratio, according to manufacturer’s protocol. The plate was incubated at 37 degrees for 1 hour, and then mounted on fluorescent reader, where fluorescence at specified time point is recorded at 560 excitation/590 emission.

### ATP assay

Cells were seeded in 96-well plate at 4,500 cells per well and grown for 16 hours. CellTiter-Glo^®^ reagent (Promega, Madison, WI) was added to each well according to the manufacturer’s protocol and the plate mounted on luminescence reader. The amount of luminescence in each well is proportional to the amount of ATP.

### Western Blot

Whole cell lysates were collected in boiling Laemmli buffer (0.125 M Tris-HCl, pH 6.8, 4% SDS, 0.13 mM bromophenol blue, 1 M sucrose) mixed with beta-mercaptoethanol (at a ratio 50:1). Lysates were run on a 10% polyacrylamide gel, transferred onto PVDF (Millipore, Billerica, MA), blocked in 10% milk in TBST (20 mM Tris, 137 mM NaCl, 0.1% Tween 20, pH 7.5) and probed with appropriate antibodies for 16 hours. Membranes were then incubated with HRP-conjugated secondary antibodies (1:5000) for one hour prior to developing using ECL-plus (Pierce, Waltham, MA). Kodak BioMax XAR film was used for chemiluminescence detection.

### Cathepsin B assay

This assay was performed as previously described [7]. Briefly, cells were plated at 80% confluency, at which time complete media was replaced with serum free media and the indicated conditions. After incubation, media was removed and cellular debris were collected by brief centrifugation. Media was then concentrated via Amicon filtration concentration devices 10,000 Daltons (Millipore). Samples were then titrated to pH 5.0–5.5 and active cathepsin B was then detected via a fluorogenic cathepsin B activity assay (Calbiochem, San Diego, CA) according to the manufacturer protocol. Simultaneously, cell lysates were extracted with RIPA buffer at 4 degrees. Protein concentration was determined using colorimetric Pierce BCA assay (Thermo Fisher Scientific). Cathepsin B activity was normalized to total cellular protein level by calculating the ratio cathepsin B activity over protein level in each condition.

### Lentiviral delivery of shRNA

shRNA directed toward Rab7 (CCGGGCCACAATAGGAGCT GACTTTCTCGAGAAAGTCAGCTCCTATTGTGGCTTTTT) was delivered into DU145 prostate cancer cell using Mission^™^ Lentiviral Transduction Particles (Sigma-Aldrich) according to manufacturer’s protocol and as previously described [6,7]. shRNA expression was maintained under puromycin (1.8 μg/mL) selection. Non Target (NT) shRNA targeting no known mammalian genes was used as a negative control (Sigma-Aldrich, shc202V).

### Transfection cells with plasmids encoding LC3-GFP-mCherry

The retroviral-based pBABE-mCherry-EGFP-LC3B plasmid (Plasmid #22418) was purchased from Addgene (Cambridge, MA). The virus was pseudotyped using the pPAM3 packaging vector after co-transfection into HEK293T cells and viral supernatants were filtered (0.2 micron), aliquoted and stored at -80C. Transfection experiments were carried out using Lipofectamine transfection reagent (Life Technologies) according to manufacturer’s protocol.

### Motility and invasion assays (IncuCyte)

96 well ImageLock Microplate (Essen Bioscience, Ann Arbor, MI) were coated with type-I collagen (Becton Dickinson, Franklin Lakes, NJ). Cells were plated and grown to 90% confluency. Identical wounds were made in each well with the 96 well wound healer (Essen Bioscience). Cells were washed to remove floating cells and debris. 20% Matrigel (Corning) was placed in wells designated for invasion studies. Treatment conditions were applied in each well. Plates were mounted on the IncuCyte Zoom imaging platform (Essen Bioscience) which is maintained at 37 degrees with 5% CO2 and acquires real-time images for the same region in each well every 4 hours. A mask was created to determine wound healing for each well. Relative Wound Density (RWD) was used to quantify tumor cell invasion and motility.

### DQ-collagen IV degradation assay

DU145 cells were grown for 2 days on coverslips that were layered with 100% matrigel (Corning) and DQ-collagen IV (Invitrogen Life Technologies, diluted at final concentration of 25 μg/mL). After treatment overnight with drug in presence or absence of HGF, cells were fixed in 4% (w/v) paraformaldehyde for 30 minutes. Next, cells were washed 2 times with PBS and incubated with Alexa Fluor 635 phalloidin (Life Technologies) diluted 1:200 in BSP for 30 minutes to stain the actin cytoskeleton. Cells were then washed 2 times with PBS and coverslips were mounted. Colonies and cleaved DQ-collagen IV were visualized on Leica TCS SP5 Laser scanning confocal microscope and images were captured using LAS AF software.

### Statistics

GraphPad Prism 5.0 and Microsoft excel software were utilized to perform the statistics. One or two-tailed Student’s t test was used to analyze statistical differences. All graphs show the mean and the standard deviation (SD). Differences at p< 0.05 were considered statistically significant. IC_50_ of the drugs was calculated using the nonlinear regression equation on GraphPad Prism. Drug screening was conducted on 2 replicates. Robust Z’ factor was calculated using the median and median absolute deviation of the control positive and negative of each plate and only plates with positive value were validated [24–27]. Z’ factor = 1 –{3(SDn + SDp)/(Mn—Mp)} [20], where Mp, Mn, SDp, SDn are mean and standard deviation of positive control (EIPA) or negative control (acid alone). Size effect of each compound was calculated using normalized percentage of inhibition (NPI). NPI = (H-xi)/(H-L) time 100; where H = mean of negative control and L = mean of positive control and xi the value of the corresponding compound. Statistical difference of the compound’s effect relative to the negative control was calculated using Z score, which is the value of each compound normalized to the negative control. Z score = (xi—H/SDn, where xi is the value of the corresponding compound, H and SDn are the mean and the standard deviation of the negative control in each plate respectively. Only drugs showing a Z score below minus 4 and NPI above 50% were re-examined for future studies.

## Results

### A novel imaging-based high content screening approach identifies drugs that inhibit anterograde lysosome trafficking

In order to identify clinically available compounds that prevent acidic pHe-mediated anterograde lysosome trafficking, we developed a high content screen using the Cellomics Array Scan IV imaging platform (Fig 1A). DU145 prostate cancer cells were seeded in 96 well plates. Low pH serum free media and EIPA were used as negative and positive controls, respectively. Compounds from four independent chemical libraries were applied at approximately 5 μM concentration to cells treated with pH 6.4 serum free RPMI overnight. Cells were fixed and lysosomes were identified with a lysosome associated membrane protein-1 (LAMP1) antibody, an established lysosomal marker, while the nuclei were visualized with DAPI. The Cellomics Arrayscan platform was used to visualize the immunofluorescence in each well using a 20x objective and a compartmental analysis software quantified lysosome distribution within each cell. Lysosome distribution was determined by analyzing the number of LAMP-1 positive puncta within the designated ‘ring’ region (Fig 1B).

Cells with lysosomes displaying JLA (EIPA treated) had fewer lysosomes within the ring region as compared to cells with lysosomes located diffuse throughout the cytoplasm. Assays were validated by calculating the Z’ factor which measures the ability of the test to differentiate between positive and negative controls [24]. The functional effect for each compound relative to controls was calculated using a “normalized percent of inhibition” score (NPI). Stronger inhibition of anterograde lysosome trafficking confers a higher NPI value. Statistical significance of the “mean ring spot” difference between compound and negative control was measured using a Z score approach (Fig 1C). Compounds with a NPI score above 50% and Z score below -4 were selected (right lower quadrant as indicated by the red arrow) and re-examined visually to eliminate false positives. Among 2210 tested compounds, eighteen drugs were identified as hits and verified by secondary screening. Many of these compounds are already known to be microtubule disrupting agents, and predictably they would block outward movement of lysosomes.

Niclosamide, an anti-helminthic agent, was also identified as one of the hits. Since, niclosamide has been demonstrated to have anti-cancer effects in glioblastoma [28], non-small cell lung cancer [29], colorectal cancer [30], and breast cancer [31], we chose to further investigate the role of niclosamide on lysosome movement in prostate cancer cells.

### Niclosamide is toxic at concentrations of 1.0 micromolar

First, we tested the cellular toxicity of niclosamide in order to identify the range of concentrations at which this drug could be used for experimental studies. Niclosamide was applied to DU145 prostate cancer cells at varying concentrations over time. Cell viability was measured by a fluorescence-based cell assay (S1A Fig). Niclosamide treatment resulted in reduced proliferation at concentrations of 0.6 μM and above by 24 and 48 hours post treatment. The decrease in cell viability became more apparent at 48 hours and at a dose of 1 μM and above when we notice a decrease in viable cells at 48 hours versus 24 hours. The half-maximal inhibitory concentration (IC_50_) was calculated as 1.01 μM (S2 Fig). Therefore, for the remainder of the experiments we performed, we chose a concentration that did not exceed 1 μM and experiments that did not exceed 24 hours.

### Niclosamide inhibits anterograde lysosome trafficking in cancer cells

**Fig 2:**
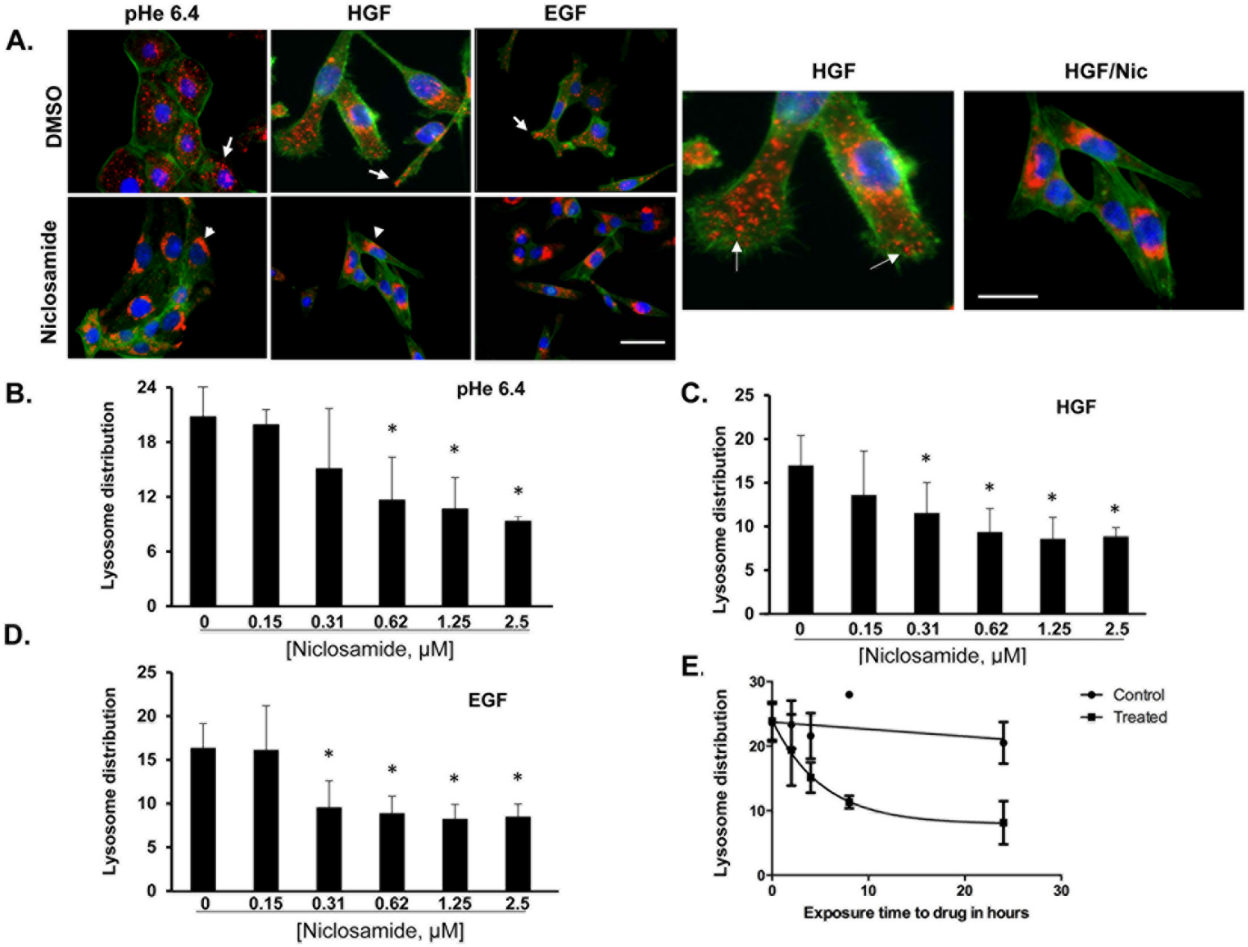
Incubation with niclosamide prevents acidic pHe and growth factor-induced lysosome trafficking. **(A)** DU145 cells were treated with pHe 6.4, 33 ng/mL HGF or 100 ng/mL EGF in the presence or absence of 0.5 μM niclosamide. Cells were fixed and stained for DAPI (blue), actin (green), and LAMP-1 (red). In control and in EGF or HGF-treated cells the lysosomes are located at the periphery (white arrows) whereas in niclosamide-treated cells the lysosomes are around the nucleus (white arrowheads). Scale bars: 10 μm. DU145 cells were treated overnight with varying concentrations of niclosamide diluted in **(B)** low pH media (pH 6.4), **(C)** media containing 33 ng/mL HGF, or **(D)** media containing 100 ng/mL EGF and relative lysosome distribution was calculated using the cellomics imager. Quantification of lysosome position is shown as the average of the calculated “mean ring spot count channel 3”. Error bars represent the SD from at least 3 independent experiments. * denotes statistical significance (p<0.01) versus niclosamide. **(E)** DU145 cells were treated with 1 μM Niclosamide over time and relative lysosome distribution was calculated using the cellomics imager. Quantification of lysosome position is shown as the average of the calculated “mean ring spot count channel 3”. Error bars represent the SD from at least 3 independent experiments.

To further characterize niclosamide as a lysosome trafficking inhibitor, DU145 prostate cancer cells were treated overnight with DMSO or niclosamide and exposed to acidic pH 6.4 media, 33 ng/ml HGF or 100 ng/ml EGF for 16 hours. Cells were then fixed and stained with DAPI (blue), LAMP1 (red), and phalloidin (green) (Fig 2A). Cells treated with DMSO displayed anterograde lysosome trafficking in response to acidic pHe, HGF and EGF. However, cells treated with niclosamide do not undergo anterograde lysosome trafficking in response to acidic pHe, HGF or EGF; instead, lysosomes remain clustered in the perinuclear region. This confirms the results of the high-content drug screen that identified niclosamide as an inhibitor of lysosome trafficking.

A dose response study was conducted to determine the minimum effective dose of niclosamide required to initiate JLA. DU145 cells were treated with varying concentrations of niclosamide for 16 hours in the presence or absence of acidic pHe (Fig 2B), HGF (Fig 2C), or EGF (Fig 2D). Cells were immunostained to detect LAMP1 and DAPI was used to identify nuclei. The relative lysosome distribution was analyzed using the Cellomics Imager. Niclosamide significantly blocked re-distribution of lysosomes at a concentration as low as 312 nM after stimulation with HGF or EGF, and at 625 nM after stimulation with acidic pHe.

Next, we carried out a time course analysis to identify the minimum amount of time that was required before niclosamide was effective at inducing JLA. DU145 cells were exposed to niclosamide over time in the presence of acidic media (pH 6.4). Fig 2E indicates that lysosome positioning was altered at soon as 2–4 hours after exposure to niclosamide, and JLA increased over time. No change in lysosome positioning was observed in vehicle control treated cells. Collectively, these data indicate that niclosamide alters the position of lysosome at a dose as low as 312 nM and as early as 2 hours.

### Niclosamide-induced JLA does not involve inhibition of ATP production

Previous work has implicated kinesin, an ATPase motor protein, in plus-end-directed lysosome movement (outward lysosomal movement) [32–34]. Since niclosamide is known for its ability to target the oxidative phosphorylation of parasites [22,23], we sought to determine whether niclosamide-induced JLA was due to ATP depletion, resulting from an inhibited oxidative phosphorylation, and consequently alteration of the activity of ATP-driven proteins such as kinesin. Accordingly, DU145 prostate cancer cells were treated with vehicle control or niclosamide over a 4 hour period of time and intracellular ATP levels, a surrogate of oxidative phosphorylation, was measured using a luminescence-based assay (S1B Fig). Although, the impact of niclosamide on lysosome positioning is observed as early as 2 hours after exposure to drug, no significant difference in ATP level was observed between the cells treated vehicle control or niclosamide. This observation indicates that niclosamide-induced JLA occurs via a mechanism that doesn’t require an alteration of oxidative phosphorylation.

### Niclosamide-induced JLA does not involve F-actin or microtubule disruption

Endocytic organelles, including lysosomes, traffic along both microtubules and actin filaments [11]. Microtubules are commonly used for rapid, long-distance lysosome movement whereas the use of actin filaments usually results in slower movement over short distances [11]. To determine whether niclosamide altered the actin cytoskeleton, DU145 cells were treated with vehicle control, cytochalasin (a disrupter of actin), or niclosamide for 2.5 hours. Cells were fixed and stained for actin (S3A Fig). Cells treated with niclosamide still displayed an intact actin cytoskeleton similar to vehicle control treated cells. However, cytochalasin D effectively disrupted the actin cytoskeleton. To test whether niclosamide altered the microtubule network, we treated cells with vehicle control, niclosamide, or nocodazole for 2.5 hours and then fixed and stained for α-tubulin (S3B Fig). Vehicle control and niclosamide-treated cells showed intact microtubules, while nocodazole-treated cells showed a collapsed microtubule network. Together, these data indicate that niclosamide does not alter the microtubule or actin cytoskeleton and that altering the cytoskeleton architecture does not account for the action of niclosamide.

### The PI3K and MAPK signaling pathways are not necessary for niclosamide to induce JLA

HGF induces the phosphorylation of the c-Met receptor, resulting in the activation of downstream effectors, including the PI3K-AKT pathway [35]. HGF also stimulates anterograde lysosome trafficking, an event that requires PI3K activity [36]. To test whether niclosamide inhibited HGF-mediated lysosome trafficking by blocking the PI3K-AKT pathway, we conducted a time course experiment where cells treated with HGF over time in the presence or absence of niclosamide. Whole cell lysates were collected and analyzed by western blot (S4A Fig). As anticipated, AKT was activated in response to HGF signaling, and this was not blocked by niclosamide. These data indicate that niclosamide is not preventing HGF/c-Met activity or the activation of critical downstream signaling pathways, and that dampened signaling is not the mechanism by which niclosamide prevents HGF-mediated anterograde lysosome trafficking.

In previous studies, we demonstrated that Tro required the mitogen activated protein kinase (MAPK) pathway to induce JLA [4]. Here, we sought to determine the signaling pathways required for niclosamide to induce JLA, employing signaling pathway specific inhibitors. DU145 cells were pretreated with the PI3K inhibitor LY294002, or the MAPK inhibitor U0126 for 30 minutes before the addition of niclosamide for 16 hours. Cells were then treated with or without acidic pHe of 6.4 the following morning for 2 hours and then fixed and stained for LAMP1. The mean lysosome distribution relative to the nucleus was calculated using the Cellomics Imager (S4B Fig). Neither the MAPK nor the PI3K pathways were required for niclosamide-stimulated JLA. Western Blot analysis indicated that these drugs prevented the phosphorylation of their target kinases (data not shown). These data indicate that niclosamide works to cluster lysosomes near the nucleus by a mechanism that is different from Tro.

### Niclosamide inhibits protease secretion and ECM degradation

**Fig 3:**
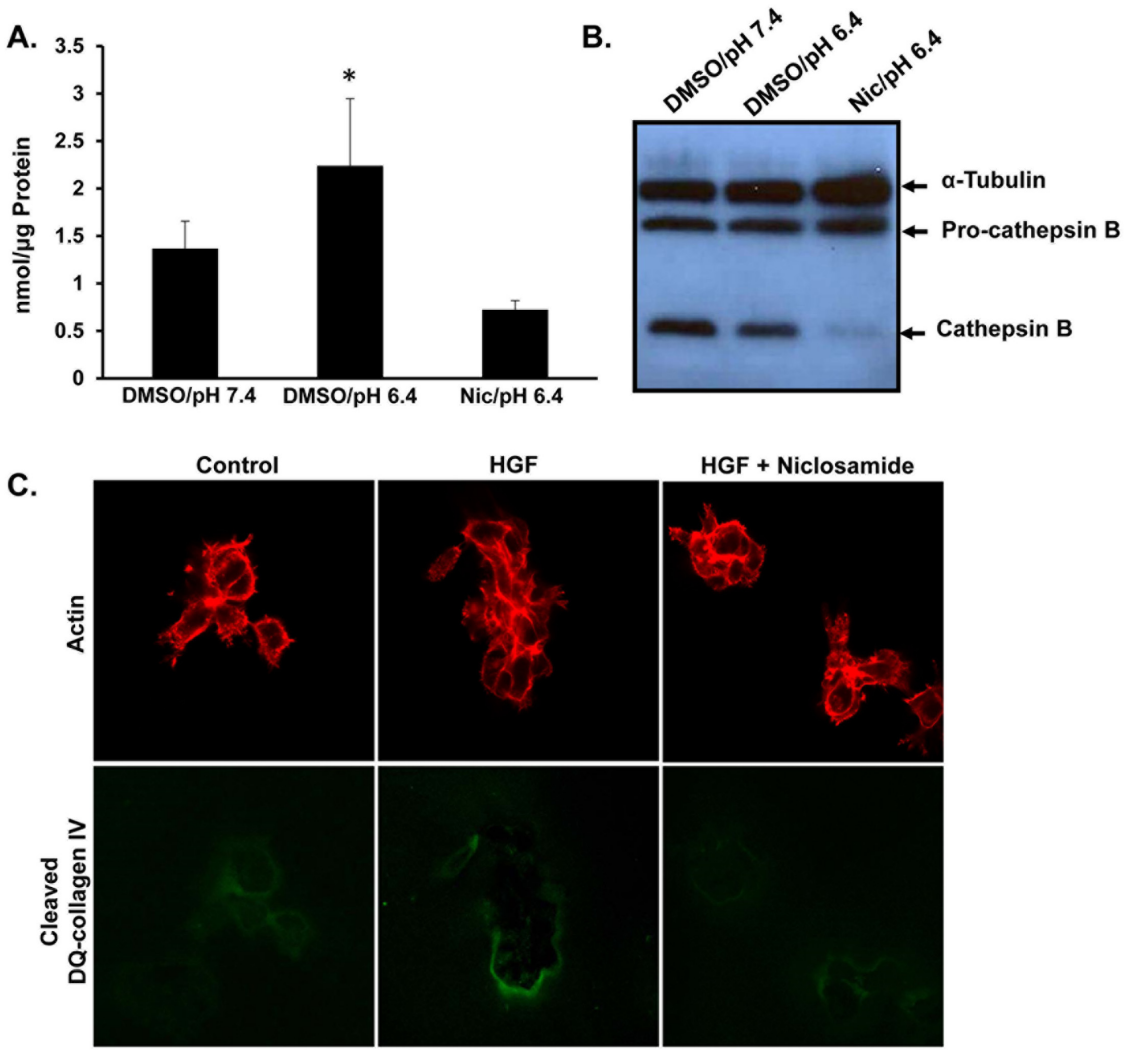
Niclosamide inhibits the acidic pHe-induced cathepsin B secretion and prevents DQ collagen IV cleavage. **(A)** DU145 cells were treated for 24 hours with DMSO in pH 7.4 media, DMSO in pH 6.4 media or 1 μM niclosamide in pH 6.4 media. The activity of secreted cathepsin B was determined. * denotes statistical significance (p<0.05) versus DMSO/pH 7.4. Error bars represent the SD from at least 3 independent experiments. **(B)** Cells were treated with DMSO in pH 7.4 media, DMSO in pH 6.4 media or 1 μM niclosamide in pH 6.4 media. Whole cell lysates were collected and Western blot analysis was performed for the indicated proteins. **(C)** DU145 cells were grown for two days on matrigel containing DQ collagen IV and then incubated for an additional 24 hours with 33 ng/mL HGF or 0.3 μM niclosamide. Cells were fixed and stained for actin (red). Green represents cleaved DQ-collagen IV. Representative confocal images are shown.

Previous work determined that treatment with acidic pHe of 6.4 was sufficient to induce lysosome trafficking which paralleled lysosome secretion, including the release of cathepsin B into the extracellular environment, a lysosomal protease that contributes to matrix degradation and tumor invasion [4,7,36–38]. EIPA and Tro both block anterograde lysosome movement and also prevent cathepsin B secretion [4]. Since lysosomes constitute a major storehouse of active cathepsin, we asked whether reduced cathepsin B secretion paralleled niclosamide-induced JLA. To determine whether levels of secreted cathepsin B correlated with blocking peripheral lysosome movement by niclosamide, DU145 cells were incubated 24 hours with pHe 7.4 medium, pHe 6.4 medium or niclosamide 1 μM in pH 6.4 media, and the activity of acidic-induced cathepsin B secreted into the culture media was quantified (Fig 3A). Incubation with acidic pH for 24 hours caused a two-fold increase in secretion of cathepsin B, and niclosamide significantly inhibited acidic pHe-induced cathepsin B secretion. Addition of niclosamide to the media collected from the condition pH 7.4 did not affect the activity cathepsin B that had already been secreted into the media, suggesting that niclosamide is not directly inhibiting the enzyme activity of this protease (data not shown). Next, we harvested whole cell lysates from DU145 prostate cancer cells treated with pH 7.4 media, pH 6.4 media, or pH 6.4 media plus niclosamide. Western blot analysis revealed a reduction in activated cathepsin B levels with no significant change in the pro-cathepsin B level compared to controls (Fig 3B). These data suggest that niclosamide prevented processing of lysosomal cathepsin B.

Remodeling of the ECM is an important step in cancer cell invasion and metastasis [39]. To analyze whether treatment with niclosamide, preventing secretion of cathepsin B and presumably other proteases, was also accompanied by a reduction in ECM degradation, we utilized a DQ-collagen IV degradation assay, as previously described [40]. DQ-collagen IV fluoresces upon proteolytic cleavage, and thus acts as visual readout for protease activity and matrix breakdown. Briefly, DU145 cells were grown on coverslips with matrigel containing DQ-collagen IV. Cells were then treated with niclosamide in the presence or absence of HGF, and then fixed and stained for phalloidin (red) following a 16 hour incubation. The green signal corresponds to cleaved DQ-collagen IV (Fig 3C). An increase in DQ-collagen IV florescence was observed in HGF-treated cultures, and this fluorescent signal was blocked upon niclosamide treatment. Together, these data indicate that niclosamide prevented the secretion of lysosomal proteases and reduced ECM degradation.

### Niclosamide blocks cancer cells invasion and motility

**Fig 4:**
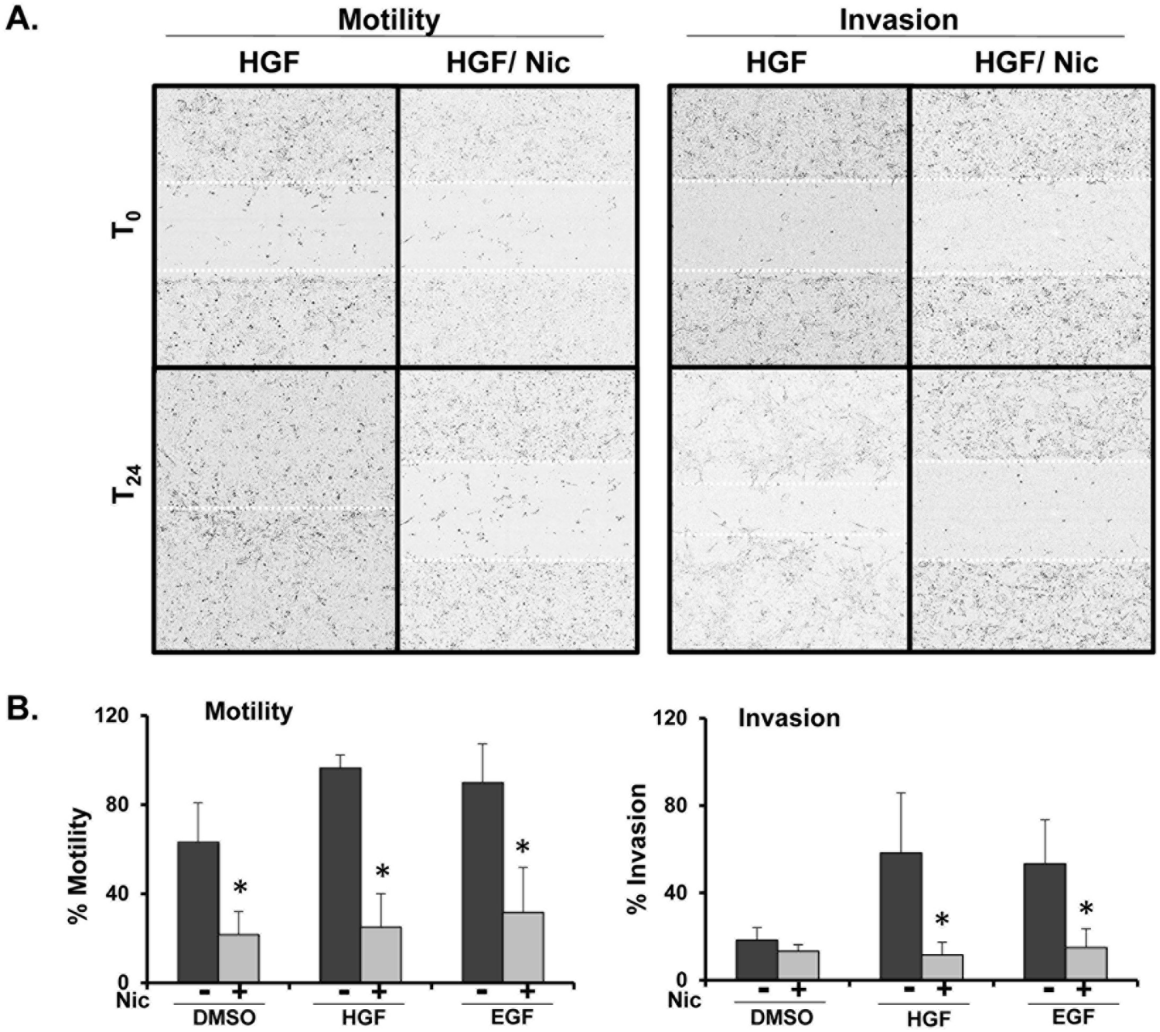
Niclosamide blocks growth factor-induced motility and invasion of DU145 prostate cancer cells. **(A)** DU145 cells were seeded in collagen-coated 96 well plates and allowed to form a confluent monolayer prior to wounding with a 96 well scratcher. For invasion assays, matrigel was added on top of the cells after wounding. Cells were allowed to migrate or invade in the presence of 33 ng/mL HGF or 100 ng/mL EGF in the presence or absence of 0.3 μM niclosamide. Wound closure was measured in real time using the IncuCyte Imager. Dotted lines indicate wound size at time 0 and 24 hours. **(B)** Motility and invasion were calculated by the IncuCyte platform as relative wound density percentage at 24 hours. Error bars represent the SD from at least 3 independent experiments. * denotes statistical significance (p<0.01) of niclosamide versus respective control.

It has been proposed that peripheral lysosome localization promotes increased cathepsin B secretion, localized extracellular matrix degradation, and tumor cell invasion [7,9,36]. To determine whether niclosamide prevents tumor cell motility and/or invasion, DU145 cells were seeded in collagen-coated 96 well plates for 48 hours, followed by creation of a wound. Matrigel was added (20% final) to the wells, thus requiring cells to “invade” through matrigel to close the wound. Cells were treated with HGF or EGF in the presence or absence of niclosamide 0.3 μM and allowed to migrate or invade into the wounded area for 24 hours. The IncuCyte platform was utilized to visualize motility and invasion of cells in real time (Fig 4A). Relative wound density percentage was used to quantify the degree of invasion and motility (Fig 4B). Similar to what we observed as regards cathepsin B secretion, HGF and EGF-induced tumor cell invasion and motility were also significantly reduced following treatment with niclosamide.

### Niclosamide blocks growth factor induced migration and invasion of tumor cells independent of Rab7 expression

We have previously demonstrated that Tro, an anti-diabetic agent, inhibited anterograde lysosome trafficking and reduced tumor invasion and inhibition was dependent on Rab7 activity [4–6]. Rab7 is a small molecular weight GTPase that recruits dynein motor complexes to late endosomes and lysosomes, thus promoting retrograde trafficking [6,12]. To test whether Rab7 was necessary for niclosamide-mediated suppression of invasion and motility, DU145 cells were transduced with lentivirus-delivered shRNA targeting Rab7 resulting in notable reduction of Rab7 protein levels (S5 inlay). Rab7KD or NT DU145 cells were treated with HGF and EGF, and motility (S5A Fig) and invasion (S5B Fig) were evaluated using the IncuCyte platform. Niclosamide treatment resulted in the reduction of HGF- and EGF-induced motility and invasion in both NT and Rab7 KD, suggesting that unlike Tro, niclosamide works independently of Rab7.

### Niclosamide-induced lysosomal perinuclear aggregation precedes autophagosomal formation

**Fig 5:**
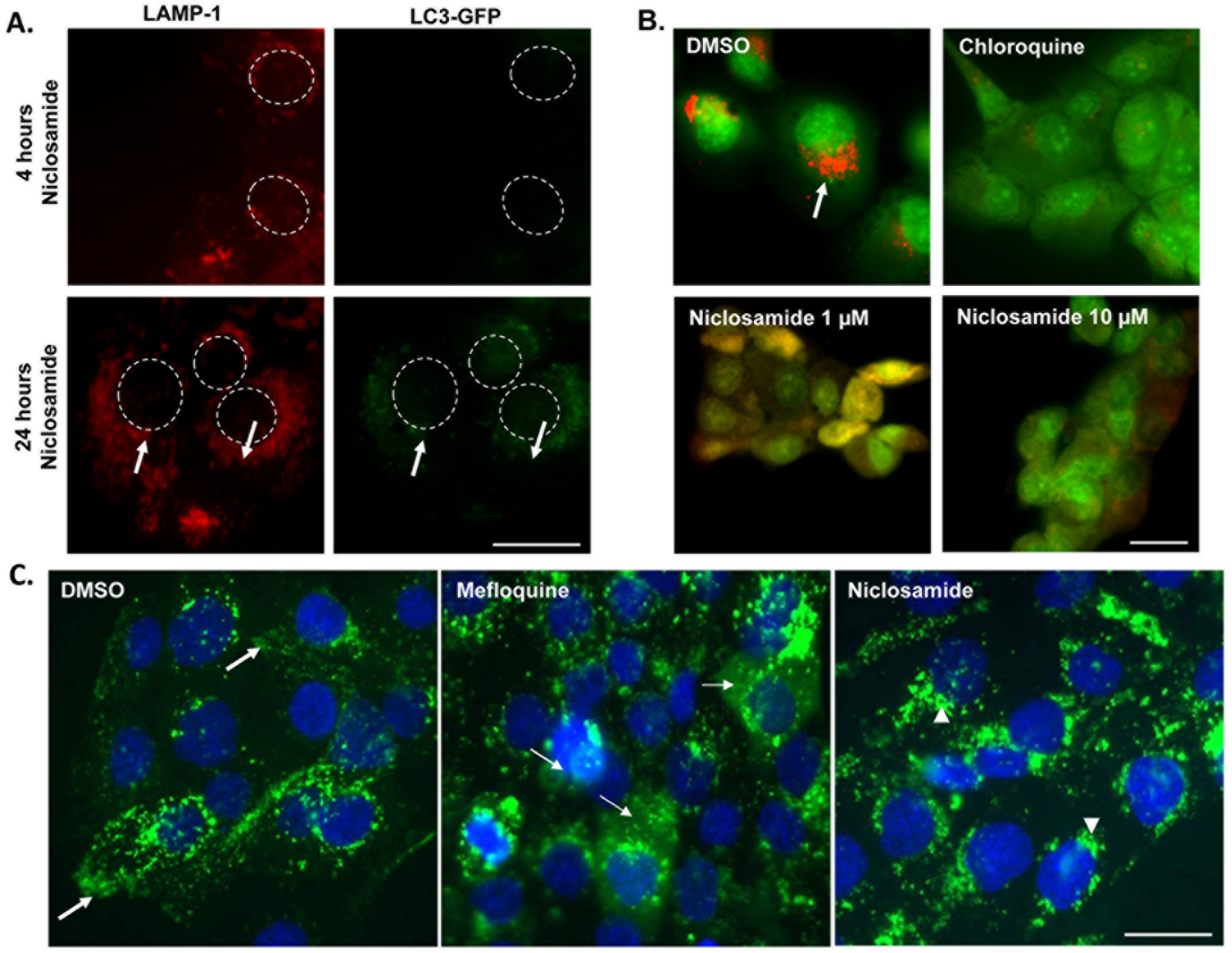
Lysosomes in niclosamide-treated cells are not leaky and are not autophagosomes. **(A)** LC3-mCherry-GFP transfected DU145 cells were treated with 1 μM niclosamide for 4 hours or 24 hours and cells were fixed and stained for LAMP-1. Dashed circles represent nuclei. Arrows indicate increased colocalization of LC3-GFP with LAMP-1. Scale bars: 10 μm. **(B)** DU145 cells were incubated with Acridine Orange, washed, and treated with DMSO, niclosamide or chloroquine for 16 hours. Red represents acridine orange accumulation in the acidic lysosomes, as seen in DMSO condition. In cells treated with chloroquine or different concentrations of niclosamide, the leaked acridine orange in cytosol is no more concentrated in the acidic lysosomal compartment and therefore its color turns into green. Scale bars: 10 μm. **(C)** DU145 cells were loaded with dextran 40kDa (green) and then treated overnight with DMSO, 10 μM mefloquine, or 0.6 μM niclosamide. Cells were fixed and stained for DAPI (blue). In control cells lysosomes have a peripheral distribution (bold arrows), whereas in niclosamide treated cells lysosomes are intact and found near the nucleus (arrowheads). Mefloquine induces lysosomal membrane permeabilization, LMP, as manifested by green cytosolic haziness (thin arrows) and is the positive control. Scale bars: 10 μm

As niclosamide has been demonstrated to induce autophagosome formation [41], we asked whether niclosamide-induced JLA was actually a result of autophagy induction. For that purpose, we analyzed the level of autophagosome formation using DU145 cells transduced with a plasmid encoding the light chain 3 (LC3) tagged with GFP and mCherry; LC3 is a marker of autophagosomes [42]. Transduced DU145 cells were treated with niclosamide in serum free media for various periods of time, and cells were fixed and stained for LAMP1 (red) (Fig 5A). At 4 hours after drug exposure, LAMP1 positive lysosomes formed aggregates around the nucleus, but no increase in puncta was observed in GFP channel, suggesting that the LAMP-1 positive vesicles were not autophagosomes. However, at 24 hours, an increase in LC3-GFP puncta co-localizing with LAMP1 was observed, suggesting that these LAMP-1 positive aggregates are autolysosomes. Taken together, these findings suggest that niclosamide-mediated peri-nuclear aggregation of lysosomes occurs prior to autophagosome formation. Since autophagosome formation occurs in the peri-nuclear region [43,44], it is possible that the lysosomal perinuclear aggregation, resulting from niclosamide treatment, promotes autophagosomes fusion with lysosomes.

### Niclosamide alters lysosomal pH, a mechanism that may mediate JLA

Lysosome acidity is necessary for the conversion of pro-cathepsin into mature cathepsin [45] and we found that niclosamide prevents the formation of mature cathepsin B (Fig 3B). Therefore, we tested whether niclosamide might reduce this process by lowering intra-lysosomal acidity. Thus, DU145 cells were loaded with acridine orange, a pH sensitive dye that accumulates in acidic vesicles. Cells were then treated with vehicle control, niclosamide, or chloroquine, a positive control known for its ability to disrupt lysosomal pH through LMP [39] (Fig 5B). We found that acridine orange accumulated in perinuclear vesicles of vehicle control treated cells, but not in cells treated with chloroquine or niclosamide, suggesting that niclosamide functions to raise the luminal lysosome pH.

Next, we measured the degree of LMP to determine if the intra-lysosomal pH neutralization associated with niclosamide was in fact due to lysosomal membrane disruption allowing proton leakage [46]. DU145 prostate cancer cells were loaded with FITC-dextran, treated with vehicle control, mefloquine known for its ability to disrupt lysosomal membrane [47], or niclosamide (Fig 5C) and subsequently visualized via immunofluorescence microscopy. A hazy dextran fluorescence in the cytosol was seen with mefloquine treatment indicating LMP. This was not observed with niclosamide, indicating that niclosamide was not inducing LMP. Unlike niclosamide, no juxtanuclear lysosomal aggregation was observed with mefloquine, pinpointing that LMP is not a required step to cause JLA.

### Increased lysosomal pH correlates with lysosome distribution

**Fig 6:**
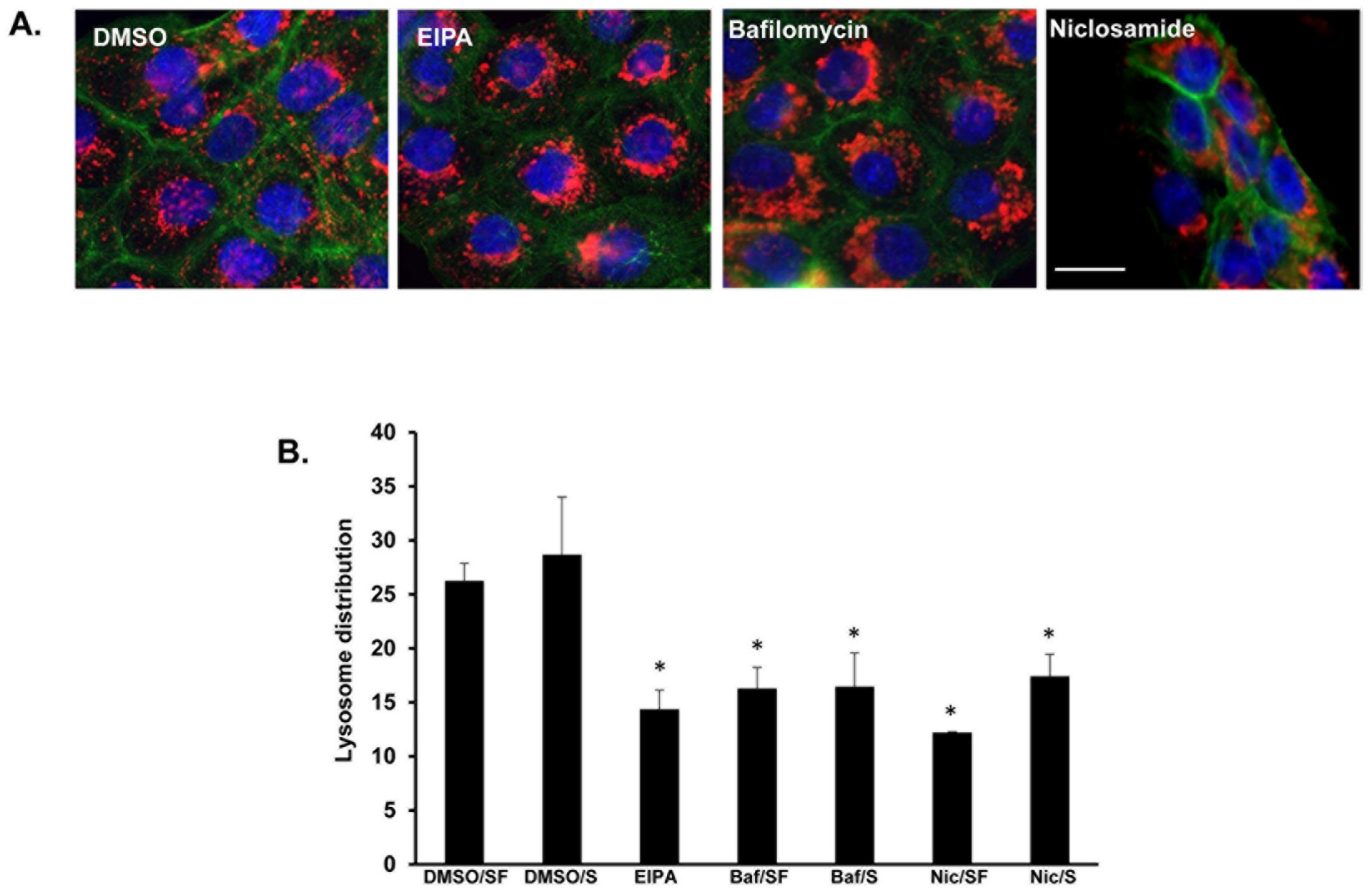
Bafilomycin A1, a specific inhibitor of vacuolar-type proton pump, induced perinuclear distribution of lysosomes. **(A)** DU145 cells were treated for 16 hours with DMSO, 25 μM EIPA, 0.1 μM bafilomycin, 1 μM niclosamide or 50 μM chloroquine. Then, pH was dropped to 6.4 in all conditions for an additional 2 hours. Cells were then fixed and immunostained for LAMP1 (red), DAPI (blue) and actin (green). Scale bars: 10 μm. **(B)** DU145 cells were treated for 16 hours with DMSO, bafilomycin A1 (Baf), niclosamide (Nic), or EIPA in serum free (SF) and complete media (S). Lysosome distribution was calculated “mean ring spot count channel 3” using the Cellomics Imager. Error bars represent the SD from at least 3 independent experiments. * denotes statistical significance (p<0.05) relative to treatment with DMSO.

Next, we hypothesized that the increase in lysosomal pH triggered by niclosamide induced lysosome movement towards the nucleus. To test this, DU145 cells were treated with EIPA (a sodium-proton exchanger inhibitor) or bafilomycin A1 (a Vacuolar type H+ ATPase inhibitor) in the presence of acidic media. Cells were fixed and stained for LAMP-1 (red), actin (green), and DAPI (blue) (Fig 6A). Perinuclear localized lysosomes were observed in cells treated with EIPA, or bafilomycin at concentrations below their IC_50_ (S2 Fig). Additionally, cells were treated with vehicle, EIPA, bafilomycin, or niclosamide in the presence or absence of serum. Lysosome distribution was analyzed using the Cellomics imager (Fig 6B). Lysosomes were located closer to the nucleus in cells where the lysosome pH was collapsed. These observations implicated that the raise in the intralysosomal pH correlated to the trafficking of lysosomes to the perinuclear region.

### Niclosamide impacts lysosome trafficking, motility, and tumor invasion in glioma cells

**Fig 7:**
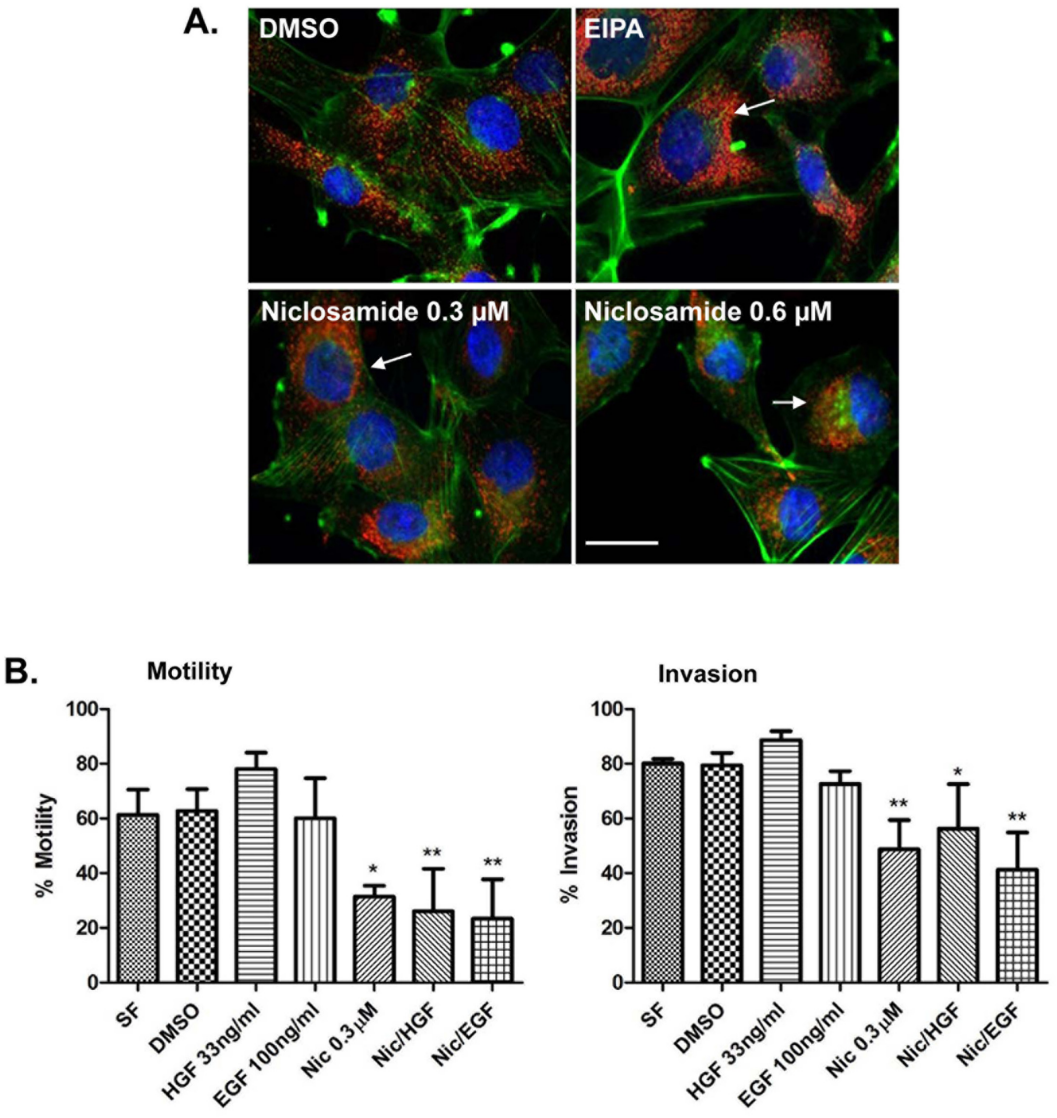
Niclosamide inhibits lysosome trafficking, motility, and invasion in glioma A172 cells. **(A)** A172 cells were treated for 8 hours with DMSO, 25 μM EIPA, or varying concentrations of niclosamide diluted in low pH media (pH 6.4). Next, cells were fixed and stained for LAMP-1 (red), actin (green) and DAPI (blue). Lysosomes in DMSO control have a peripheral location whereas in niclosamide or EIPA-treated cells they are located around the nucleus (as indicated by arrows). Scale bars: 10 μm. **(B)** A172 cells were plated in collagen-coated 96 well plates and allowed to form a confluent monolayer prior to wounding. Next, 20% matrigel was added to the wells for which invasion is to be studied. Cells were allowed to migrate or invade in the presence of DMSO, 33 ng/mL HGF or 100 ng/mL EGF in the presence or absence of 0.3 μM niclosamide. Motility and invasion were calculated by IncuCyte Imager and the relative wound density percentage at 24 hours post wound is shown. Error bars represent the SD from at least 3 independent experiments. * p<0.05 treatment versus serum free or DMSO, ** p<0.01 treatment versus serum free or DMSO.

In order to determine whether the niclosamide-mediated lysosome trafficking and motility and invasion phenotypes were general phenomena, we examined niclosamide’s effect in A172 human glioma cells. Gliomas are highly invasive brain cancers and their invasiveness is dependent on lysosome trafficking toward the plasma membrane [37,38]. Therefore, we tested whether niclosamide altered lysosome positioning in A172 glioma cells. A172 cells were treated with vehicle control, EIPA, or varying concentrations of niclosamide in the presence of acidic media (pH 6.4). Cells were then fixed and stained for LAMP-1 (red), DAPI (blue), or actin (green) (Fig 7A). JLA was observed in cells treated with EIPA and niclosamide, indicating that niclosamide caused lysosome redistribution in glioma cells. Next, we asked whether niclosamide inhibited the growth factor-mediated motility or invasion of glioma cells. To test this, A172 cells were grown in a confluent monolayer and wounded. For invasion assays, matrigel was plated over the cells. Cells were then stimulated to move with HGF or EGF in the presence or absence of niclosamide. Wound closure indicative of motility or invasion was calculated using the IncuCyte imaging system (Fig 7B). Niclosamide treatment significantly inhibited HGF and EGF-mediated invasion and motility. Collectively, these data indicate that niclosamide causes JLA and blocks invasion and motility in multiple cancer cell lines and that niclosamide is not acting in a cell line or tissue specific manner.

## Discussion

Aberrant lysosome trafficking has been described in several types of cancer. The Sloane group found that altered lysosomal locations correlated with increased grade of invasive brain cancer, and later showed that cathepsin B released from these more peripheral lysosomes played a role in tumor invasion [9,40,48]. More recently, Tu et al showed that cathepsin B delivered to podosomes by lysosomes was involved in matrix degradation [10]. We have demonstrated that the acidic microenvironment and different growth factors secreted in the tumor reactive stroma contribute to the anterograde lysosome trafficking, and tumor cell invasion [5–7,36]. Pharmacologic inhibitors of sodium-proton exchangers, such as EIPA and Troglitazone, have been shown to be very effective in preventing anterograde lysosome trafficking, thereby decreasing protease secretion and cell invasion [4,7,36]. The effect of these 2 drugs on lysosome positioning and tumor invasion was abrogated following Rab7 depletion, establishing a cause-effect relationship between peripheral movement of lysosomes and enhanced invasiveness of tumor cells [4,6,7,36]. Based on these findings, regulating the position of lysosomes emerges as a possible target for future development of drugs that reduce cancer invasion. EIPA and Tro cannot be advanced into the clinic owing to their toxic profiles [20]. Thus, we have designed an imaging-based high throughput assay to screen for repositioned drugs, already used in clinic for non-cancer indications, which prevent lysosome trafficking. We identified niclosamide, a human anti-helminthic drug, as one of 18 “hits” that is a potent inducer of JLA and an inhibitor of tumor cell motility, invasion, and protease secretion.

Our drug screen uses Cellomics compartmental analysis software that calculates the number of spots in a pre-set intracellular virtual mask. Our methodology proved to be valid, reproducible, and able to carry out large-scale screening because it is automated, rapid, and inexpensive, and its measurements are not affected by operator. Our screening method carries another major advantage: since most of the screened compounds are or were used for non-cancer indication and have good safety profile, many of the hit drugs can be advanced with more ease from the bench into the clinic for testing. The pharmaceutical industry is facing a wide array of challenges in drug development including rising in cost, increasing regulation, heightened concerns about drug safety, growing competition from generic companies, and an increasing gap between stagnant product output and increase in research and development cost. These challenges have pushed many pharmaceutical companies to adopt alternative strategies such as drug repurposing, which implicates repositioning of existing drugs for new indications. Drug repurposing carries several advantages including the potential of reduction of the cost and the amount of time spent by exploring drugs of well-known safety profile.

In this project, we identified niclosamide as an inhibitor of low pHe and growth factor- induced anterograde lysosome trafficking at a concentration as low as 0.3–0.6 μM. We have also demonstrated the potential therapeutic activity of niclosamide against prostate cancer cells at the level of tumor invasion and extracellular matrix degradation. This effect was detected in our model with concentration that is achievable clinically, and below the peak concentration normally seen after single oral ingestion of the drug [23]. These findings indicate that niclosamide is unlikely to be toxic within the therapeutic range that elicits anti-tumor invasion activity.

Further dissection of the mechanism of action of niclosamide showed that this drug does not impact or require tubulin, actin, or the MEK and PI3 kinase pathways to re-distribute lysosomes to the perinuclear region, and its action was independent from its effect on mitochondrial oxidative phosphorylation, a known mechanism of action of antihelminthics. These data suggest that niclosamide is acting to induce JLA by a mechanism different from other known drugs that also cause JLA.

Secretion of lysosomal protease accounts for ECM proteolysis that facilitates tumor invasion. Our study demonstrated that niclosamide reduced acidic-triggered cathepsin B secretion, an event thought to be important for tumor invasion. In order to determine whether this is secondary to reduced export or production of cathepsin B, we measured the intracellular cathepsin level. By analyzing the whole cell lysates of cells treated with niclosamide or low pH media, we have found that the ratio of cathepsin B/ pro-cathepsin B is lower in niclosamide condition. This suggest that a decrease in cathepsin B secretion may be in part due to reduced processing of pro-cathepsin into mature cathepsin B caused perhaps by the alteration in intra-lysosomal acidity by the drug. Indeed, low pH is critical for the optimal activity of lysosomal protease [9]. Our results are concordant with previous experiments showing that niclosamide possesses the ability to dissipate protons from lysosomes to the cytosol [49]. However, reduced lysosomal content exocytosis as a mechanism by which niclosamide decreases cathepsin B secretion remains a viable possibility.

In previous reports, induction of autophagy was accompanied by formation of auto-phagosomes around the nucleus [43,44]. We report that the aggregation of lysosomes around the nucleus precedes the formation of auto-phagosome in LC3-mCherry-GFP cells (Fig 5C). Interestingly, inducing autophagy by means other than niclosamide (treating cells with rapamycin) does not alter lysosome positioning (data not shown). Furthermore, cells treated with bafilomycin, a drug that inhibits the fusion lysosomes and autophagosomes, exhibit an aggregation of lysosomes around the nucleus (Fig 6A). These two observations support the notion that the formation of auto-phagosomes and perinuclear aggregation of lysosomes are independent from each other, at least in early stages of autophagy. It is possible that the increased number of lysosomes at the perinuclear region will potentially facilitate at a later stage their fusion with lysosomes, and as a result can control the rate of autophagosomal degradation.

Intra-lysosomal pH is determined by a balance between different components that include primarily the vacuolar-type ATPase proton pump, other ions transporters such as the chloride/proton anti-porter, ClC-7 transporter, proton leakage outside the lysosomes, and luminal buffer power [50,51]. Our data revealed that niclosamide impairs intra-lysosomal acidity without apparent disruption of lysosomal membrane, indicating that the drug may impair lysosomal acidification through a mechanism other than proton leakage across a permeable lysosomal membrane. Interestingly, EIPA an inhibitor of Na+/H+ exchanger or bafilomycin A1, a selective inhibitor of the Vacuolar type H+ ATPase pump [52], also prevented anterograde lysosome trafficking, supporting the concept that V-ATPase may play a role in outward lysosomal movement. Although EIPA is primarily an inhibitor of the sodium proton exchanger present at cellular membrane, it can indirectly lessen the activity of V-ATPase pump through reducing intra-cytosolic chloride, which will eventually elevate the amount of positive charge inside the lysosome, impeding the V-ATPase to transport proton against the electrical barrier [52]. In support of this, there is emerging evidence that various subunits of V-ATPase can regulate vesicular endocytic and exocytic trafficking. Hurtado-Lorenzo et al. described interaction of V-ATPase with small GTPase in an endosomal-acidification dependent manner to regulate endocytic pathway. Yang et al. showed that depletion of Ac45, a specific subunit of V-ATPase, impaired lysosomal exocytosis, a phenomenon that was not due to defective V-ATPase [17–19]. Taken together, one possible interpretation of niclosamide’s effect on lysosome trafficking is that it may act through similar mechanism to EIPA or bafilomycin by inhibiting directly or indirectly the V-ATPase pump machinery. Consistent with that possibility, mefloquine, an LMP inducer, didn’t affect the spatial distribution of lysosomes in our model, indicating that it is the inhibition of lysosomal proton pump, and not the defect in intra-lysosomal acidification, that promoted JLA. Future works will investigate the complex crosstalk between different NHEs, V-ATPase and their contribution to spatial lysosome distribution through interplay with other motor proteins and other regulators of vesicular trafficking. Additionally, it will aim to determine if this interaction is dependent on V-ATPase activity and/or endosomal acidification.

Niclosamide is an anti-helminthic drug that recently has shown a myriad number of activities against cancer. Recent papers have indicated numerous anti-cancer activities for niclosamide including: 1) niclosamide down-regulated Wnt signaling and elicited antitumor effect on colon cancer cell lines [30], 2) niclosamide down-regulated S100 A4, a calcium binding protein involved in metastatic process, and blocked S100 A4-mediated metastatic progression in colon cancer [53], 3) niclosamide inhibited proliferation of ovarian cancer cell lines by disrupting multiple metabolic pathways involved in biogenetics, biogenesis, and redox regulation [54], 4) niclosamide inhibited androgen receptor variants expression and overcomes enzalutamide resistance in castrate resistant prostate cancer [55], 5) niclosamide inhibited Stat3 activity, 6) niclosamide inhibited multiple signaling pathways in cancer stem cells [56] and 7) niclosamide decreased invasion of glioblastoma multiforme [28] although the mechanism for this was not revealed. Interestingly, we demonstrated that niclosamide prevents lysosome trafficking in multiple cancer cell lines, including glioma cells A172 glioma cells and the androgen refractory DU145 prostate cancer cells. We speculate that many of the mechanisms of action of niclosamide that seem so disparate on the surface may be tied to a common action, inhibition of lysosome trafficking. This carries some clinical relevance since brain tumors have tendency to invade diffusely into the surrounding brain tissue, accounting for its clinical manifestations. Currently, data regarding the clinical safety profile of niclosamide in long-term usage is lacking, and we propose to investigate whether this drug enhances the activity of radiation and/or standard antineoplastic agents used for the treatment of prostate cancer and gliomas.

In conclusion, we developed a reproducible and valid imaging-based high throughput screen used to identify drugs that block anterograde lysosome trafficking and could be used as potential anti-cancer therapy. In addition, we identified niclosamide as a hit drug that prevents anterograde lysosome trafficking and investigated the pathways required for its action. Overall, our data highlight the therapeutic potential of niclosamide in the treatment of prostate cancer and other malignancies.

## Supporting Information

S1 Fig Niclosamide inhibits the proliferation of DU145 WT at a dose above 1 μM and does not affect ATP levels. **(A)** DU145 cells were treated with varying concentrations of niclosamide for 1, 24 and 48 hours. Cell viability was calculated using Cell Titer Blue, FL 560/590. Error bars represent SD from 3 independent experiments. **(B)** DU145 cells were treated with niclosamide 1 μM or DMSO over time. The level of ATP was measured using luminescence-based assay. Error bars represent SD from 3 independent experiments.
(TIF)

S2 Fig Cell growth inhibition by niclosamide, EIPA and Bafilomycin A1. DU145 cells were treated at different drugs concentrations. The IC_50_ of niclosamide, EIPA and Bafilomycin A1 was 1.01 μM, 97.75 μM and 4.81 μM respectively. X-axis values represent log10 of each drug concentration. Y-axis values represent the ratio of cell viability at 24 hours relatively to 1 hour after drug exposure, as determined by CellTiter blue.
(TIFF)

S3 Fig Niclosamide has no effect on actin or microtubules. **(A)** DU145 cells were treated with DMSO or 1 μM niclosamide for 4 hours. Cytochalasin D was used as a control to depolymerize actin filaments. Cells were fixed and stained for actin (green) and DAPI (blue). Arrows indicate that the same cellular components (filamentous actin-arrowhead, cortical actin- closed arrow, focal adhesion- open arrow) are similar between control and niclosamide. Scale bars: 20 μm. **(B)** DU145 cells were treated with DMSO or 1 μM niclosamide for 4 hours. Nocodazole was used as a control to depolymerize microtubules. Cells were fixed and stained for α-tubulin (green) and DAPI (blue).
(TIF)

S4 Fig PI3kinase and MAPK are not required for niclosamide to prevent acidic media induced outward lysosome movement. **(A)** Cells were stimulated with 33 ng/mL HGF in the presence or absence of 0.5 μM niclosamide over time. Cell lysates were collected and Western blot analysis was performed for the indicated proteins. **(B)** DU145 cells were pre-treated with PI3K inhibitor, LY294002, or MAPK inhibitor, U0126, prior to the addition of niclosamide 1 μM for 16 hours. Cells were fixed and stained for LAMP-1 and mean lysosome distribution relative to the nucleus was calculated using the Cellomics imager. Quantification of lysosome distribution is shown as the average of relative position to the nucleus. * denotes statistical significance (p<0.05) relative to same treatment in serum free. Error bars represent the SD from at least 3 independent experiments.
(TIF)

S5 Fig Niclosamide blocks growth factor-induced motility and invasiveness independently from Rab7 status. DU145 NT and Rab7 KD cells were grown in 96 well plates and wounded with the 96 well wound healer prior to the addition of matrigel in the wells designed for invasion. Cells were allowed to **(A)** migrate or **(B)** invade in the presence of 33 ng/mL HGF or 100 ng/mL EGF in the presence or absence of 0.3 μM niclosamide. Motility and invasion were calculated using the IncuCyte platform and the relative wound density percentage at 24 hours post-wounding. Error bars represent the SD from at least 3 independent experiments. * denotes statistical significance (p<0.01) of niclosamide versus respective control.
(TIF)

## Abbreviations

(EGF): Epidermal Growth Factor
(HGF): Hepatocyte Growth Factor
(pHe): extracellular pH
(JLA): Juxtanuclear lysosome aggregation
(LMP): lysosome membrane permeabilization
(ECM): extracellular matrix
(RILP): Rab interacting lysosomal protein
(Tro): Troglitazone
(EIPA): 5-(N-ethyl-N-isopropyl)-amiloride
(LAMP1): lysosome associated membrane protein-1
(MAPK): mitogen activated protein kinase
