# Correction: A Novel High Content Imaging-Based Screen Identifies the Anti-Helminthic Niclosamide as an Inhibitor of Lysosome Anterograde Trafficking and Prostate Cancer Cell Invasion

**DOI:** 10.1371/journal.pone.0151718

**Published:** 2016-03-14

**Authors:** Magdalena L. Circu, Samantha S. Dykes, Jennifer Carroll, Kinsey Kelly, Floyd Galiano, Adam Greer, James Cardelli, Hazem El-Osta

The images for Supporting Information files S3 Fig, S4 Fig, and S5 Fig are incorrectly switched. S3 Fig is incorrect. The image that appears as S3 Fig should be S4 Fig. The image that appears as S4 Fig should be S5 Fig. The figure legends appear in the correct order. Please view the correct S3, S4, and S5 Figs as well as their legends here.

Additionally, there are errors in the legend for S3 Fig, “Niclosamide has no effect on actin or microtubules.” DU145 cells were treated with DMSO or 1 μM niclosamide for 2.5 hours rather than 4 hours. The complete, correct S3 Fig legend is shown below.

## Supporting Information

S3 Fig Niclosamide has no effect on actin or microtubules. **(A)** DU145 cells were treated with DMSO or 1 μM niclosamide for 2.5 hours. Cytochalasin D was used as a control to depolymerize actin filaments. Cells were fixed and stained for actin (green) and DAPI (blue). Arrows indicate that the same cellular components (filamentous actin-arrowhead, cortical actin- closed arrow, focal adhesion- open arrow) are similar between control and niclosamide. Scale bars: 20 μm. **(B)** DU145 cells were treated with DMSO or 1 μM niclosamide for 2.5 hours. Nocodazole was used as a control to depolymerize microtubules. Cells were fixed and stained for α-tubulin (green) and DAPI (blue).
(TIFF)

S4 Fig PI3kinase and MAPK are not required for niclosamide to prevent acidic media induced outward lysosome movement. **(A)** Cells were stimulated with 33 ng/mL HGF in the presence or absence of 0.5 μM niclosamide over time. Cell lysates were collected and Western blot analysis was performed for the indicated proteins. **(B)** DU145 cells were pre-treated with PI3K inhibitor, LY294002, or MAPK inhibitor, U0126, prior to the addition of niclosamide 1 μM for 16 hours. Cells were fixed and stained for LAMP-1 and mean lysosome distribution relative to the nucleus was calculated using the Cellomics imager. Quantification of lysosome distribution is shown as the average of relative position to the nucleus. * denotes statistical significance (p<0.05) relative to same treatment in serum free. Error bars represent the SD from at least 3 independent experiments.
(TIF)

S5 Fig Niclosamide blocks growth factor-induced motility and invasiveness independently from Rab7 status. DU145 NT and Rab7 KD cells were grown in 96 well plates and wounded with the 96 well wound healer prior to the addition of matrigel in the wells designed for invasion. Cells were allowed to **(A)** migrate or **(B)** invade in the presence of 33 ng/mL HGF or 100 ng/mL EGF in the presence or absence of 0.3 μM niclosamide. Motility and invasion were calculated using the IncuCyte platform and the relative wound density percentage at 24 hours post-wounding. Error bars represent the SD from at least 3 independent experiments. * denotes statistical significance (p<0.01) of niclosamide versus respective control.
(TIF)
