# Supplementary figures and images for: A Novel High Content Imaging-Based Screen Identifies the Anti-Helminthic Niclosamide as an Inhibitor of Lysosome Anterograde Trafficking and Prostate Cancer Cell Invasion
Source: PLoS One. 2016 Jan 19;11(1):e0146931. doi: 10.1371/journal.pone.0146931 (PMC4718621; doi:10.1371/journal.pone.0146931)

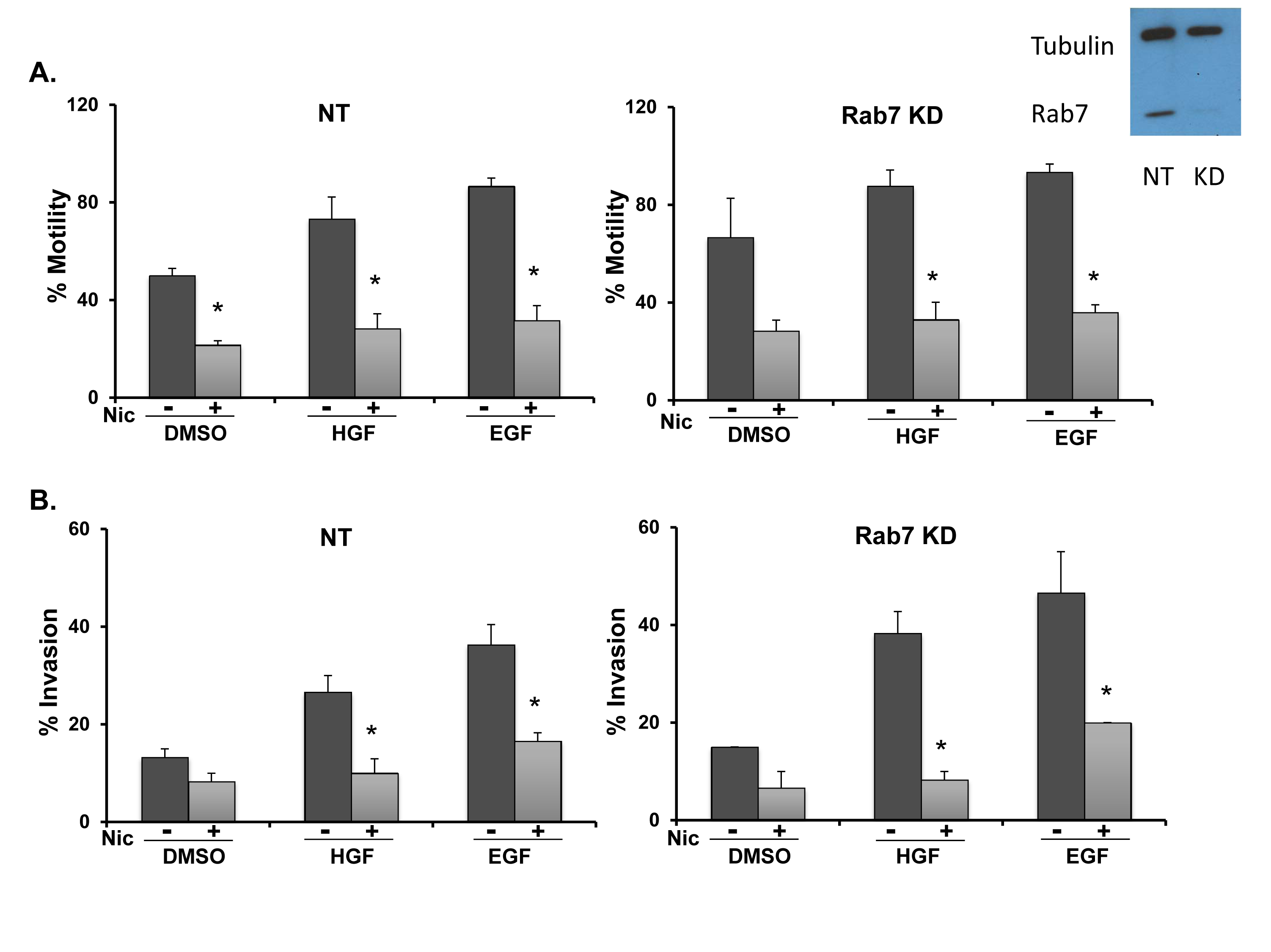

Supplement: S5 Fig — DU145 NT and Rab7 KD cells were grown in 96 well plates and wounded with the 96 well wound healer prior to the addition of matrigel in the wells designed for invasion. Cells were allowed to (A) migrate or (B) invade in the presence of 33 ng/mL HGF or 100 ng/mL EGF in the presence or absence of 0.3 μM niclosamide. Motility and invasion were calculated using the IncuCyte platform and the relative wound density percentage at 24 hours post-wounding. Error bars represent the SD from at least 3 independent experiments. * denotes statistical significance (p<0.01) of niclosamide versus respective control. (TIF) [file pone.0146931.s005.tif]
