# Supplementary figures and images for: Correction: A Novel High Content Imaging-Based Screen Identifies the Anti-Helminthic Niclosamide as an Inhibitor of Lysosome Anterograde Trafficking and Prostate Cancer Cell Invasion
Source: PLoS One. 2016 Mar 14;11(3):e0151718. doi: 10.1371/journal.pone.0151718 (PMC4790951; doi:10.1371/journal.pone.0151718)

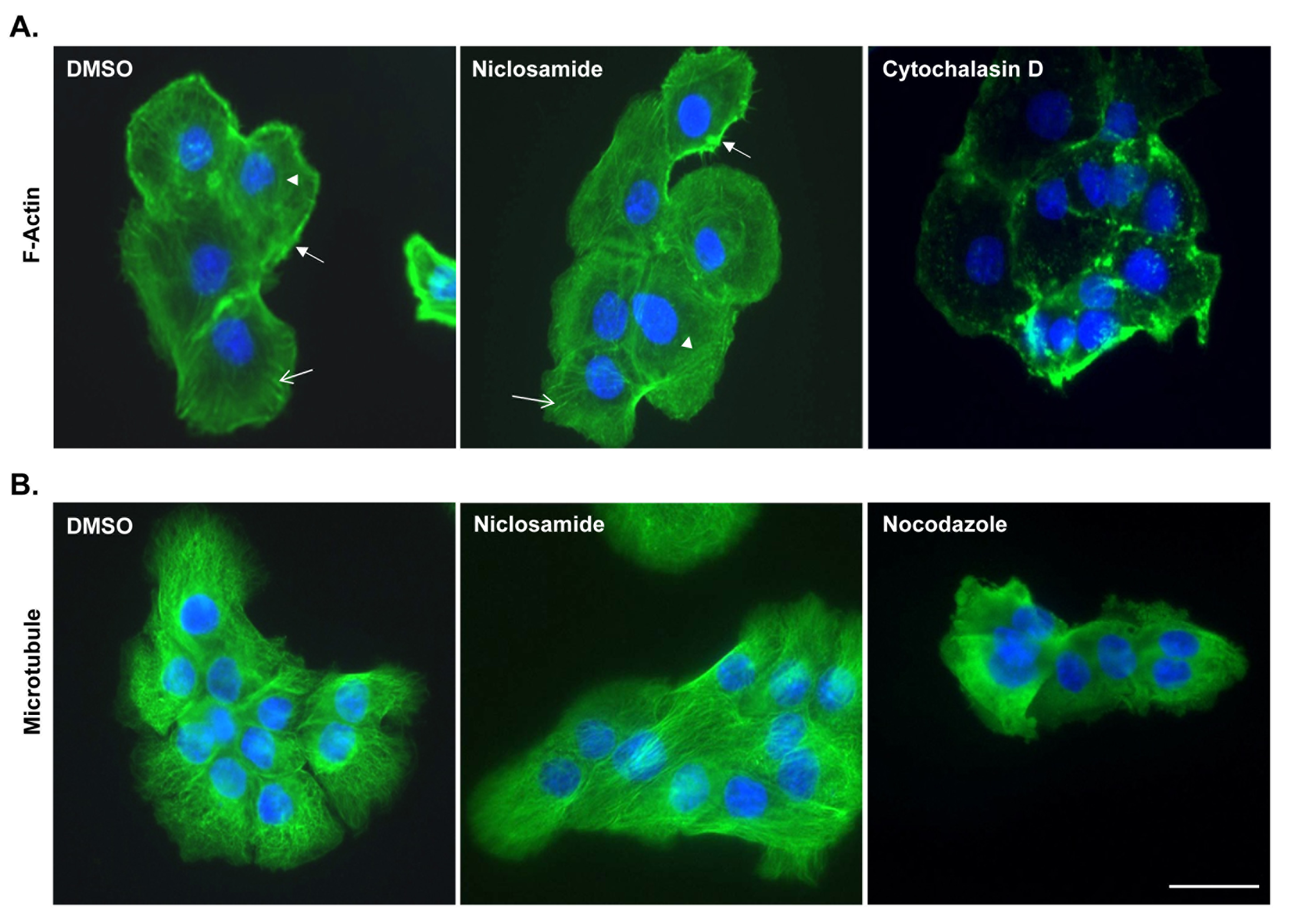

Supplement: S3 Fig — (A) DU145 cells were treated with DMSO or 1 μM niclosamide for 2.5 hours. Cytochalasin D was used as a control to depolymerize actin filaments. Cells were fixed and stained for actin (green) and DAPI (blue). Arrows indicate that the same cellular components (filamentous actin-arrowhead, cortical actin- closed arrow, focal adhesion- open arrow) are similar between control and niclosamide. Scale bars: 20 μm. (B) DU145 cells were treated with DMSO or 1 μM niclosamide for 2.5 hours. Nocodazole was used as a control to depolymerize microtubules. Cells were fixed and stained for α-tubulin (green) and DAPI (blue). (TIFF) [file pone.0151718.s001.tiff]
